# Supplementary material for: Mitigation of SARS-CoV-2 transmission at a large public university
Source: Nat Commun. 2022 Jun 9;13:3207. doi: 10.1038/s41467-022-30833-3 (PMC9184485; doi:10.1038/s41467-022-30833-3)
Supplement: Supplementary file 1 — Supplementary Information [file 41467_2022_30833_MOESM1_ESM.pdf]

Supplementary Figure Legends

Supplementary Fig. 1. The effect of different mitigation interventions on the basic reproduction number R_0 as computed in our agent-based model assuming that 60% agents are compliant with testing, isolation and quarantine. If R_0 is greater than one (orange dashed line at R_0=1), the epidemic grows exponentially. Mean R_0 values (n=10) are indicated for each conditions tested. Error bars represent SEM.

Supplementary Fig. 2. Effect of delays in contact tracing on estimated total number of infections and the peak size of quarantined population. Heat maps for the expected number of infected cases and maximal quarantined agents by the end of the semester as a function of delayed time to notify contacts (x-axis) and the fraction of contacts that is untraceable (y-axis). Delays in notification aggregate from delays in isolation of the index case, delays in completion of case investigation, and delays in establishing a successful contact with the exposed individual. The fraction of untraceable contacts needs to be small (<30%) in order for contact tracing to be effective. Delays of more than 1 day to notify the results and contacts can lead to multifold increase in epidemic size and quarantined population. For example, notifying positive cases within 1 day of positive result and instantaneous contact tracing lead to 1000 infected agents. A two-day delay in notifying the positive cases and for 30% of untraceable contacts, the total number of infected agents may increase by tenfold.

Supplementary Fig. 3. Limit of Detection (LoD) for assessment of SARS-CoV-2 nucleic acid from saliva, comparing the initial protocol performed on QuantStudio 3 **(a)** to the covidSHIELD protocol performed either on QuantStudio 7Flex **(b)** or QuantStudio 7Pro **(c)**. Saliva in 50mL conical tubes was spiked with the indicated amount of gamma-irradiated SARS-CoV-2 prior to heating at 95°C for 30 minutes. Samples were processed using the covidSHIELD assay. PCR plates were run on 3 different QuantStudio models together with a positive control (pos; SARS-CoV-2 positive control, 5.0×10^3 copies/mL) and a negative control (neg; water). Data in quadruplicates were analyzed for SARS-CoV-2 *ORF1ab* (blue triangle), *N*-gene (orange square), and *S*-gene (green circle), and MS2 (open circle). MS2 bacteriophage was added to the PCR reaction mix as internal control. Undetermined Ct values are plotted as ND. The LoD was set at the lowest concentration at which 2 out of 3 viral target genes were detected. LoD experiments were performed at least five times in different machines.

Supplementary Fig. 4. Saliva samples could be stored at room temperature for at least 7 days prior to heating and analysis without loss of sensitivity. Saliva from a SARS-CoV-2 negative subject was collected in 50 mL conical tubes. Sample was divided into sets of aliquots (one set for the negative samples and one for the positive sample). The positive samples were created by spiking the saliva with γ-irradiated SARS-CoV-2 at 5.0×10^3 viral copies/mL. Samples were further split into smaller groups for storage at either room temperature (25°C) or at 4°C at different time points. Following the incubation period, all samples were processed using the covidSHIELD assay, and together with a positive control (pos; SARS-CoV-2 positive control, 5.0×10^3 copies/mL) and a negative control (neg; water), were directly analyzed by RT-qPCR in triplicates for SARS-CoV-2 *ORF1ab* (blue triangle), *N*-gene (orange square), and *S*-gene (green circle), and MS2

(open circle). MS2 bacteriophage was added to the PCR reaction mix as internal control. Undetermined Ct values are plotted at 0. Saliva stability experiment prior to heat inactivation was repeated twice.

Supplementary Fig. 5. LOD of direct saliva-to-RT-qPCR SARS-CoV-2 nucleic acid detection using CDC-approved primers and probes. γ-irradiated SARS-CoV-2 was spiked into fresh human saliva (SARS-CoV-2 negative) in 1X Tris-Borate-EDTA buffer (TBE) at $1.0x10^2$, $5.0x10^2$, $1.0x10^3$, $2.5x10^3$, $5.0x10^3$, $1.0x10^4$, and $5.0x10^4$ viral copies/mL. Samples were incubated at 95°C for 30 min. Virus-spiked saliva samples, a positive control (pos; SARS-CoV-2 positive control, $5.0x10^3$ copies/mL) and a negative control (neg; water) were directly analyzed by RT-qPCR, in triplicate, for SARS-CoV-2 N1 gene (**a**) and N2 gene (**b**), and the human RP gene (**c**). Undetermined Ct values are plotted at 0. LoD experiments using CDC primers were performed at least three times.

Supplementary Fig. 6. Comparison of covidSHIELD processing method (**a**, heat at 95°C for 30 minutes followed by TBE/tween addition) to SalivaDirect protocol (**b**,proteinase K treatment followed by heating at 95°C for 5 minutes). γ-irradiated SARS-CoV-2 was spiked into fresh human saliva (SARS-CoV-2 negative). All samples were analyzed with the ThermoFisher TaqPath COVID-19 combo kit on a QS7 instrument in triplicates together with a positive control (pos; SARS-CoV-2 positive control, $5.0x10^3$ copies/mL) and a negative control (neg; water). MS2 bacteriophage was added to the PCR reaction mix as internal control. This experiment was performed twice.

Supplementary Fig. 7. Distribution of time interval between two consecutive tests. The distribution has a mean of 4.14 days and standard deviation of 3.4 days. The probability of a test interval larger than 7 days was 0.11. 62% of the tests were spaced at 3.5 days or less. This suggests that about 60% of the campus population was highlighly compliant with the testing protocol while the rest was somewhat compliant and only a small fraction was significantly out of compliance (inter-test time > 7 days).

Supplementary Fig. 8. 7-day rolling average for daily positive cases as computed using the agent-based model assuming different levels of compliance with isolation and quarantine compared to the confirmed cases detected using covidShield test as of October 15, 2020. (**a**) Trajectory of cases assuming an average rate of imported infections = 2 cases/day, (**b**) Trajectory of cases assuming an average rate of imported infections = 6 cases/day, and (**c**) Trajectory of cases assuming an average rate of imported infections = 10 cases/day. Compliance of the cohort of agents with high social activity levels (e.g., those who attend parties and go to bars) was assumed to be 0% prior to the temporary restrictions (essential activities) period. During the essential activity period, simulated agents are allowed to go to classes, restaurants, libraries, and coffee shops but not to attend bars and parties. Imported infections are introduced to account for incidental infections in the university population due to interaction with the surrounding community which has its own prevalence. The results suggest that the large increase in cases in the beginning of the semester may be explained as a combination of lack of compliance with isolation and quarantine and high levels of imported infections (> 10 cases/day, Panel **c**). The essential activities period helped contain the spread and led to rapid decline in cases. The model estimates

that student compliance during this period was relatively high ranging between 60%-80% (2 daily imported infections, Panel **a**), 80% (6 daily imported infections, Panel **b**), and close to 100% (10 daily imported infections, Panel **c**). After the temporary restrictions ended, the inferred compliance levels, required to match the observed cases, varied but was estimated to be between 40% and 60% with the lower end more probable if imported infections = 2 cases/day and the upper end is more probable if imported infections = 10 cases/day. Between 08/15/2020 and 10/01/2020, and assuming only 25%-50% of covid cases were detected and reported at the state level, the estimated 7-day prevalence in the state of Illinois ranged between 0.22% and 0.33%. For the socially active agent population (approximately 15,000 agents), this corresponds to imported infections ranging between 4.7 cases/day and 9 cases/day consistent with the estimates in Panels **b** and **c**. These results suggest that compliance levels with isolation and quarantine were close to 60%, the essential activities period was successful in quickly limiting the spread and higher level of compliance during that period further assisted with this, and community transmission as measured by imported infection is an important confounder in shaping the epidemic curve.

Supplementary Fig. 9. Estimating the effective exponential decay rate that was achieved by Shield during different intervals of Fall 2021. (**a**) Daily new cases by date of sample collection. (**b**) The natural logarithm of the 7-day averaged daily new cases. The slope of the dashed line *g* measures the effective exponential decay rate in the intervals over which cases were decreasing. The exponentail decary rate ranged between g = -0.126/day (halving time = 5.5.days) to g = -0.057/day (halving time = 12.1 days). Assuming a serial time interval that is gamma distributed with a mean = 4 days and standard deviation = 3.25 days, these decay rates correspond to an effective reproduction number[48] that ranges between 0.55 and 0.78. These estimates are consistent with the full time-dependent trajectories displayed in Figure 3 in the main text which demonstrate that R_t reached as low as 0.5, and was frequently around 0.75-0.85.

Supplementary Fig. 10. Measured correlations of the number of 7-day averaged daily new cases between residents in Champaign County, faculty/staff, and undergraduate students for the period between August 15 and December 23. All points in three scatter plots are colored according to their dates, as shown in the color bar on the right. (**a**) The number of 7-day averaged daily new cases of residents in Champaign county strongly correlated with that of faculty/staff especially for the period after October 18 (Pearson correlation coefficient 0.86, p-value 7.99×10^{-38} calculated using a 2-tailed test). (**b**) At the beginning of the semester (around August 31) when there was a spike in daily new cases from undergraduate students, it had little influence on the faculty/staff. While at the late period of the semester (after October 18) when the case positivity in Illinois increased, they showed a correlation (Pearson correlation coefficient 0.88, p-value 1.33×10^{-21} calculated using a 2-tailed test). (**c**) The initial spike in the number of daily new cases of undergraduate students doesn’t correlate with that of residents in Champaign County. While at the late period of the semester (after October 18), as the number of positive cases in Champaign county increased and then decreased, the number of cases of undergraduate students also showed a similar trend.

Supplementary Tables

Supplementary Table 1. Comparison of Ct values from 25 clinical samples that were split into two aliquots upon receipt, one set was processed using our covidSHIELD assay and the other set was subjected to RNA extraction using MagMax Viral/Pathogen II (MVP II) Nucleic Acid Isolation Kit (ThermoFisher).

Supplementary Table 2. Comparison of method of loading to the 384-well PCR plate (manual vs. multichannel robot) and the method of loading heat-inactivated saliva samples to the 96-deep-well plates pre-loaded with 2xTBE/1% Tween-20 buffer (manual vs. Span8 robot).

Supplementary Table 3. Endogenous & Exogenous Interference testing results for covidSHIELD assay

Supplementary Table 4. Qualitative outcome of parallel testing of paired mid-turbinate swabs and saliva with the Abbott RealTime SARS-CoV-2 assay and covidSHIELD.

Supplementary Table 5. Method comparison study completed to support the correlation between saliva samples processed with covidSHIELD and nasal samples processed with Abbott RealTime SARS-CoV-2 assay performed on the Abbott m2000 System.

Supplementary Table 6. Details to Capture on Case Report Form

Supplementary Table 7. Comparison of mid-turbinate (MT) swab and saliva from 17 individuals identified with low viral load based on MT swab analyzed using Abbott Alinity RT-PCR.

Supplementary Table 8. Quanta emission rates, and ventilation rates in different zone types. How many zones in each type is given in the “Count” column.

Supplementary Table 9: Assumption for how long agents stay in different zones.

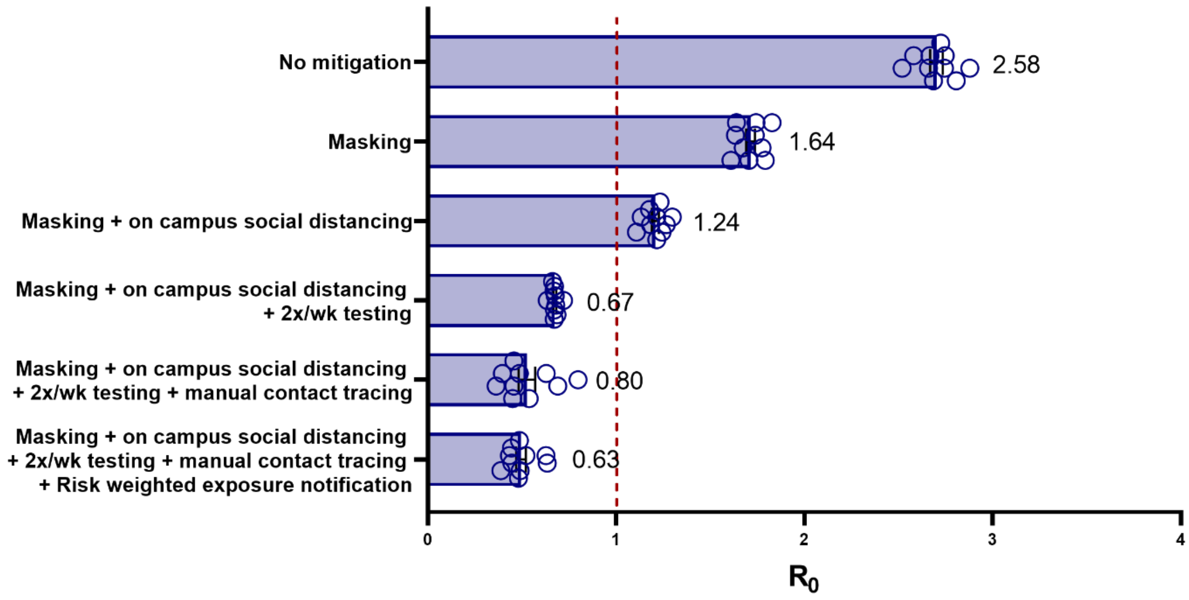

Supplementary Fig. 1. The effect of different mitigation interventions on the basic reproduction number R0 as computed in our agent-based model assuming that 60% agents are compliant with testing, isolation and quarantine. If R0 is greater than one (orange dashed line at R0=1), the epidemic grows exponentially. Mean R0 values (n=10) are indicated for each conditions tested. Error bars represent SEM.

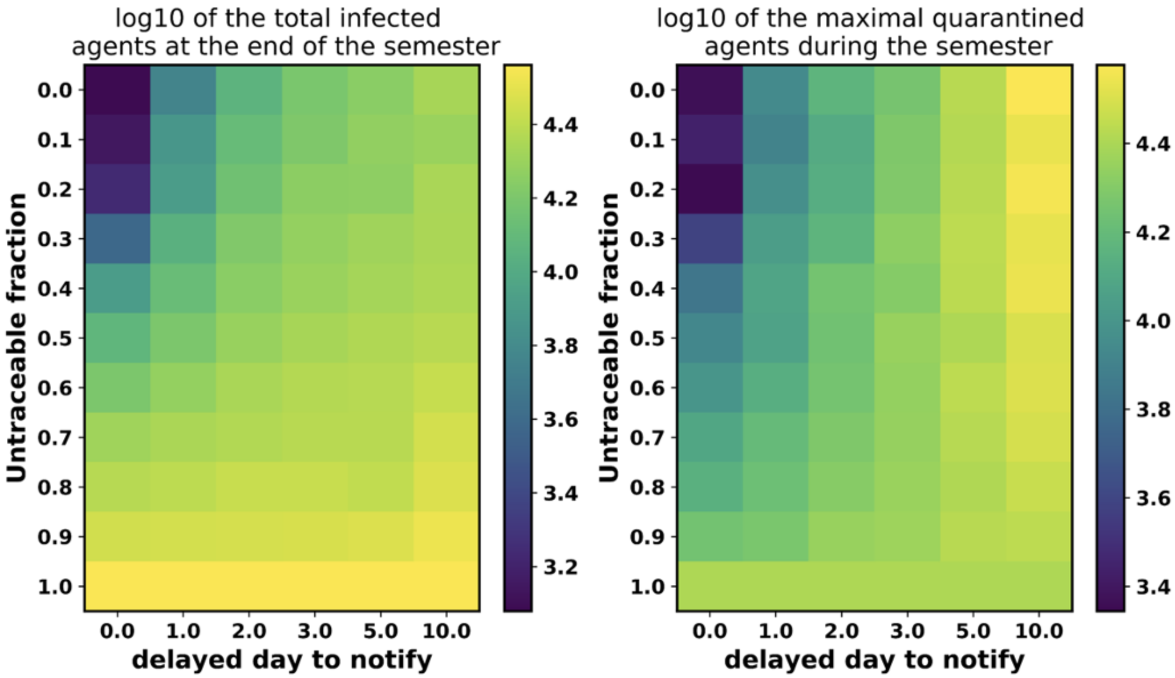

Supplementary Fig. 2. Effect of delays in contact tracing on estimated total number of infections and the peak size of quarantined population. Heat maps for the expected number of infected cases and maximal quarantined agents by the end of the semester as a function of delayed time to notify contacts (x-axis) and the fraction of contacts that is untraceable (y-axis). Delays in notification aggregate from delays in isolation of the index case, delays in completion of case investigation, and delays in establishing a successful contact with the exposed individual. The fraction of untraceable contacts needs to be small (<30%) in order for contact tracing to be effective. Delays of more than 1 day to notify the results and contacts can lead to multifold increase in epidemic size and quarantined population. For example, notifying positive cases within 1 day of positive result and instantaneous contact tracing lead to 1000 infected agents. A two-day delay in notifying the positive cases and for 30% of untraceable contacts, the total number of infected agents may increase by tenfold.

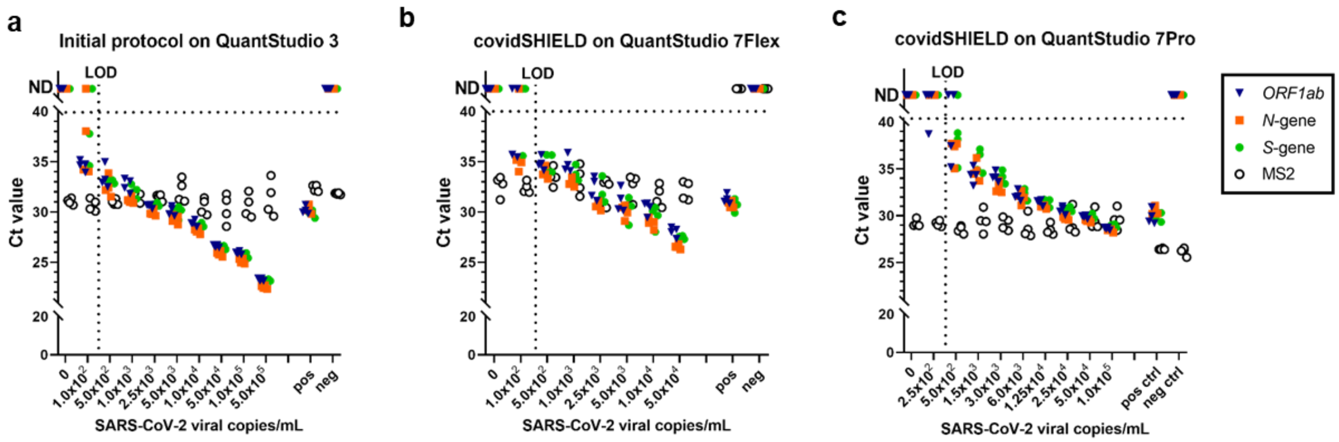

Supplementary Fig. 3. Limit of Detection (LoD) for assessment of SARS-CoV-2 nucleic acid from saliva, comparing the initial protocol performed on QuantStudio 3 (**a**) to the covidSHIELD protocol performed either on QuantStudio 7Flex (**b**) or QuantStudio 7Pro (**c**). Saliva in 50mL conical tubes was spiked with the indicated amount of gamma-irradiated SARS-CoV-2 prior to heating at 95°C for 30 minutes. Samples were processed using the covidSHIELD assay. PCR plates were run on 3 different QuantStudio models together with a positive control (pos; SARS-CoV-2 positive control, 5.0x10^3 copies/mL) and a negative control (neg; water). Data in quadruplicates were analyzed for SARS-CoV-2 *ORF1ab* (blue triangle), *N*-gene (orange square), and *S*-gene (green circle), and MS2 (open circle). MS2 bacteriophage was added to the PCR reaction mix as internal control. Undetermined Ct values are plotted as ND. The LoD was set at the lowest concentration at which 2 out of 3 viral target genes were detected. LoD experiments were performed at least five times in different machines.

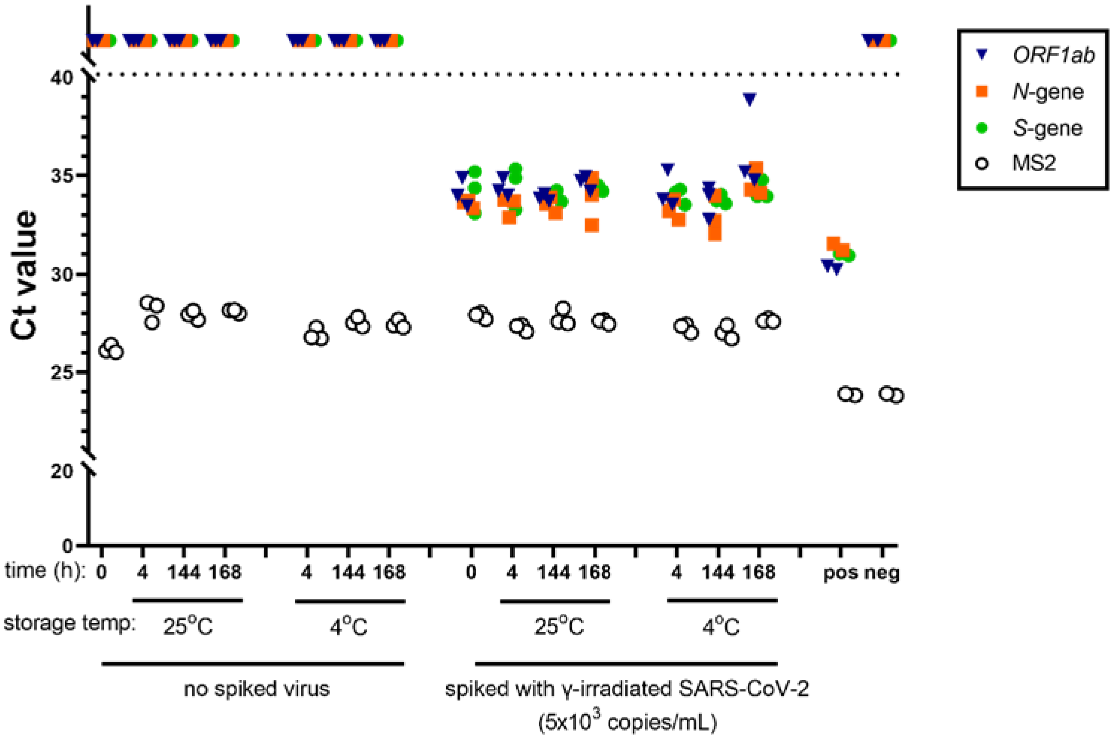

Supplementary Fig. 4. Saliva samples could be stored at room temperature for at least 7 days prior to heating and analysis without loss of sensitivity. Saliva from a SARS-CoV-2 negative subject was collected in 50 mL conical tubes. Sample was divided into sets of aliquots (one set for the negative samples and one for the positive sample). The positive samples were created by spiking the saliva with γ-irradiated SARS-CoV-2 at 5.0×10^3 viral copies/mL. Samples were further split into smaller groups for storage at either room temperature (25°C) or at 4°C at different time points. Following the incubation period, all samples were processed using the covidSHIELD assay, and together with a positive control (pos; SARS-CoV-2 positive control, 5.0×10^3 copies/mL) and a negative control (neg; water), were directly analyzed by RT-qPCR in triplicates for SARS-CoV-2 *ORF1ab* (blue triangle), *N*-gene (orange square), and *S*-gene (green circle), and MS2 (open circle). MS2 bacteriophage was added to the PCR reaction mix as internal control. Undetermined Ct values are plotted as ND. Saliva stability experiment prior to heat inactivation was repeated twice.

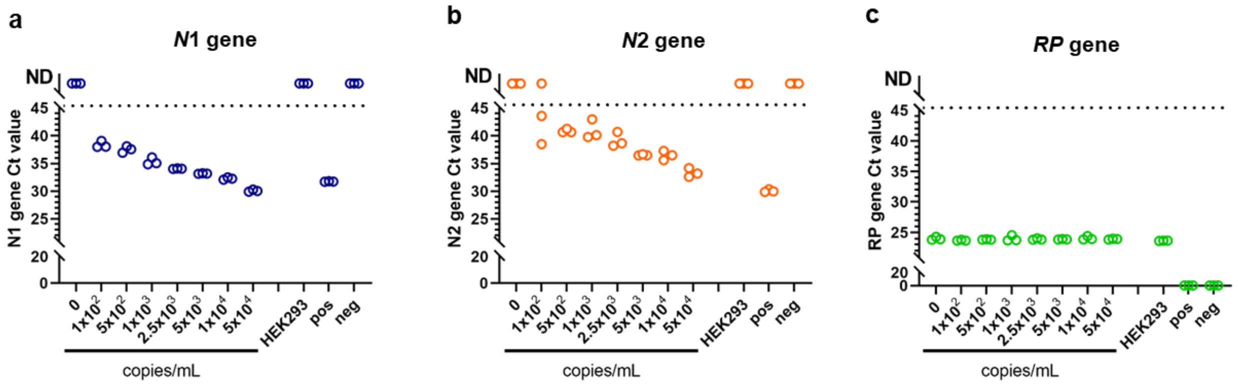

Supplementary Fig. 5. LOD of direct saliva-to-RT-qPCR SARS-CoV-2 nucleic acid detection using CDC-approved primers and probes. γ-irradiated SARS-CoV-2 was spiked into fresh human saliva (SARS-CoV-2 negative) in 1X Tris-Borate-EDTA buffer (TBE) at $1.0x10^2$, $5.0x10^2$, $1.0x10^3$, $2.5x10^3$, $5.0x10^3$, $1.0x10^4$, and $5.0x10^4$ viral copies/mL. Samples were incubated at 95°C for 30 min. Virus-spiked saliva samples, a positive control (pos; SARS-CoV-2 positive control, $5.0x10^3$ copies/mL) and a negative control (neg; water) were directly analyzed by RT-qPCR, in triplicate, for SARS-CoV-2 *N*1 gene (**a**) and *N*2 gene (**b**), and the human RP gene (**c**). Undetermined Ct values are plotted as ND. LoD experiments using CDC primers were performed at least three times.

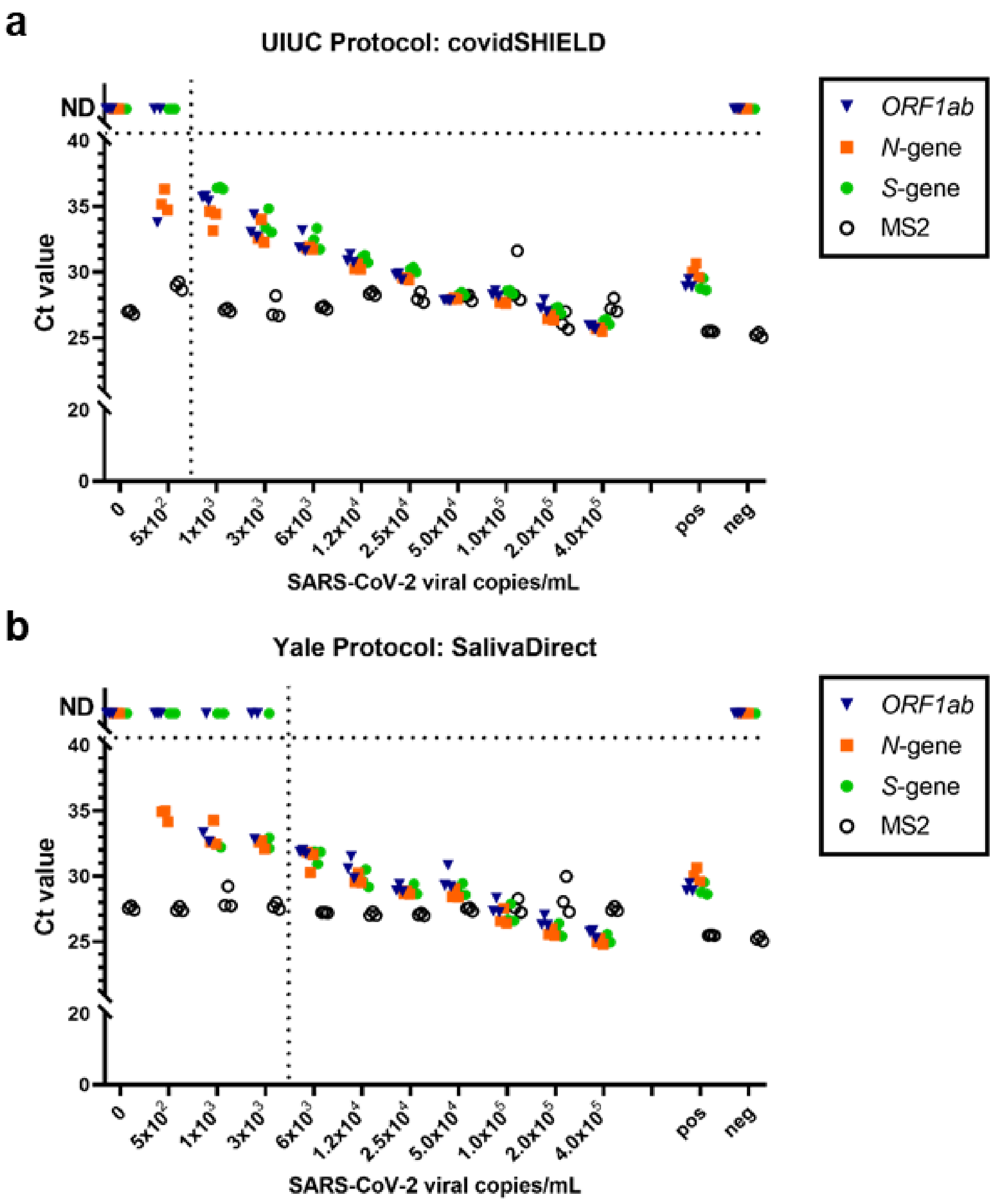

Supplementary Fig. 6. Comparison of covidSHIELD processing method (**a**, heat at 95°C for 30 minutes followed by TBE/tween addition) to SalivaDirect protocol (**b**,proteinase K treatment followed by heating at 95°C for 5 minutes). γ-irradiated SARS-CoV-2 was spiked into fresh human saliva (SARS-CoV-2 negative). All samples were analyzed with the ThermoFisher TaqPath COVID-19 combo kit on a QS7 instrument in triplicates together with a positive control (pos; SARS-CoV-2 positive control, $5.0x10^3$ copies/mL) and a negative control (neg; water). MS2 bacteriophage was added to the PCR reaction mix as internal control. This experiment was performed twice.

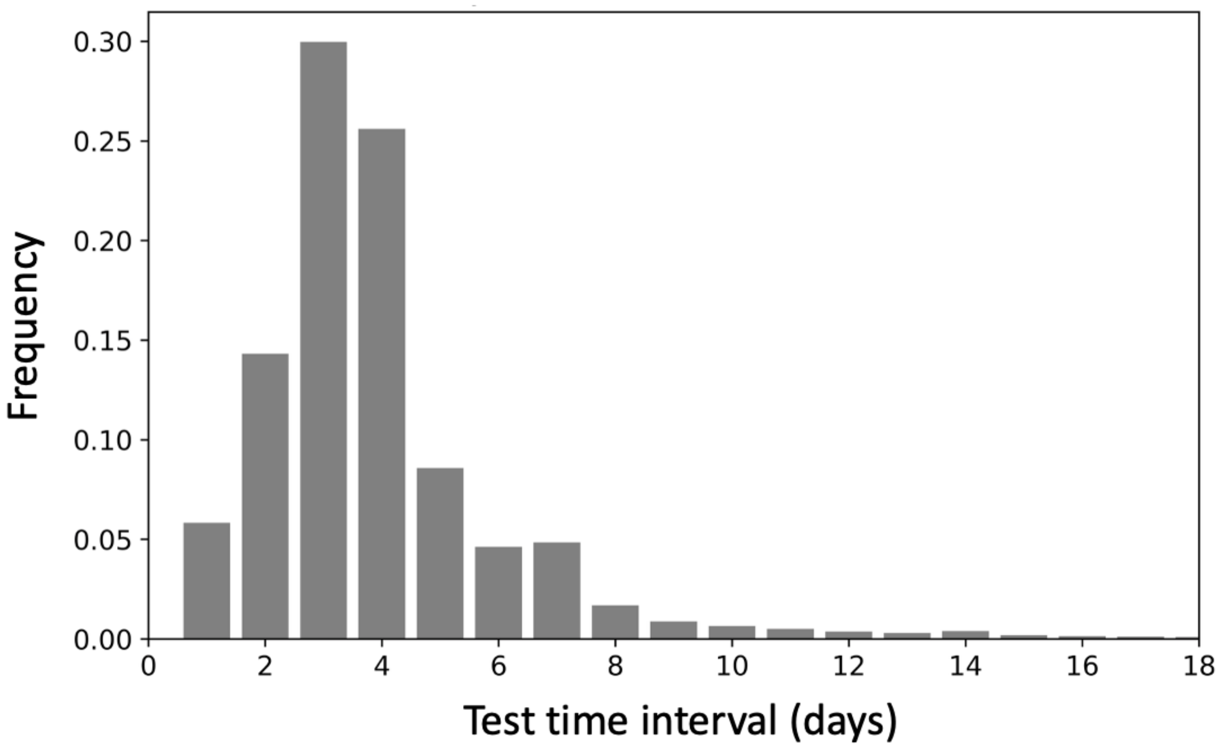

Supplementary Fig. 7. Distribution of time interval between two consecutive tests. The distribution has a mean of 4.14 days and standard deviation of 3.4 days. The probability of a test interval larger than 7 days was 0.11. 62% of the tests were spaced at 3.5 days or less. This suggests that about 60% of the campus population was highly compliant with the testing protocol while the rest was somewhat compliant and only a small fraction was significantly out of compliance (inter-test time > 7 days).

a

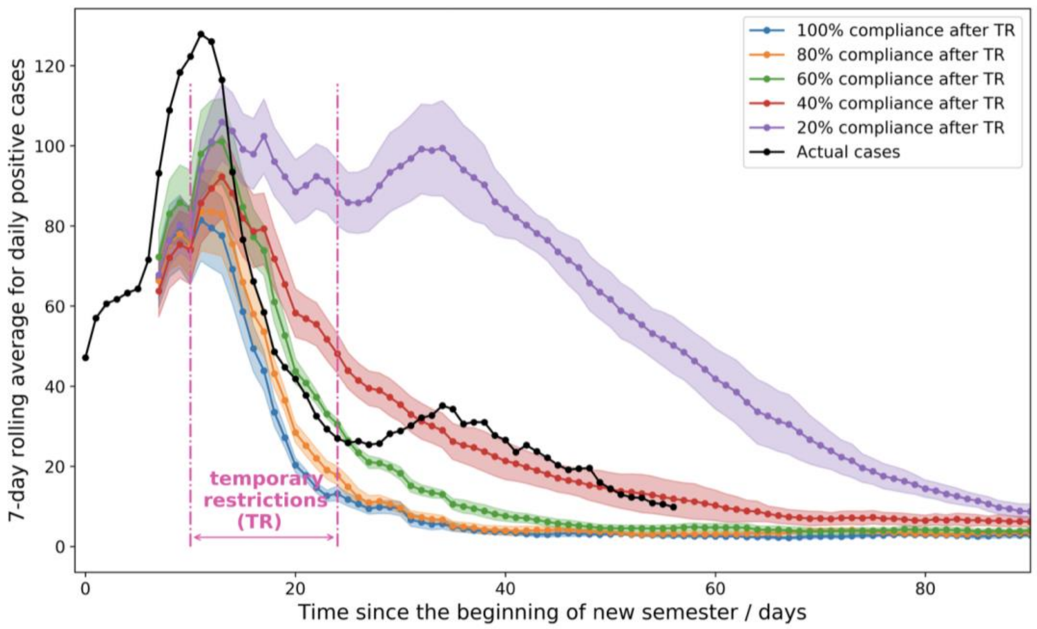
100% compliance after TR
80% compliance after TR
60% compliance after TR
40% compliance after TR
20% compliance after TR
Actual cases
temporary restrictions (TR)
7-day rolling average for daily positive cases
Time since the beginning of new semester / days

b

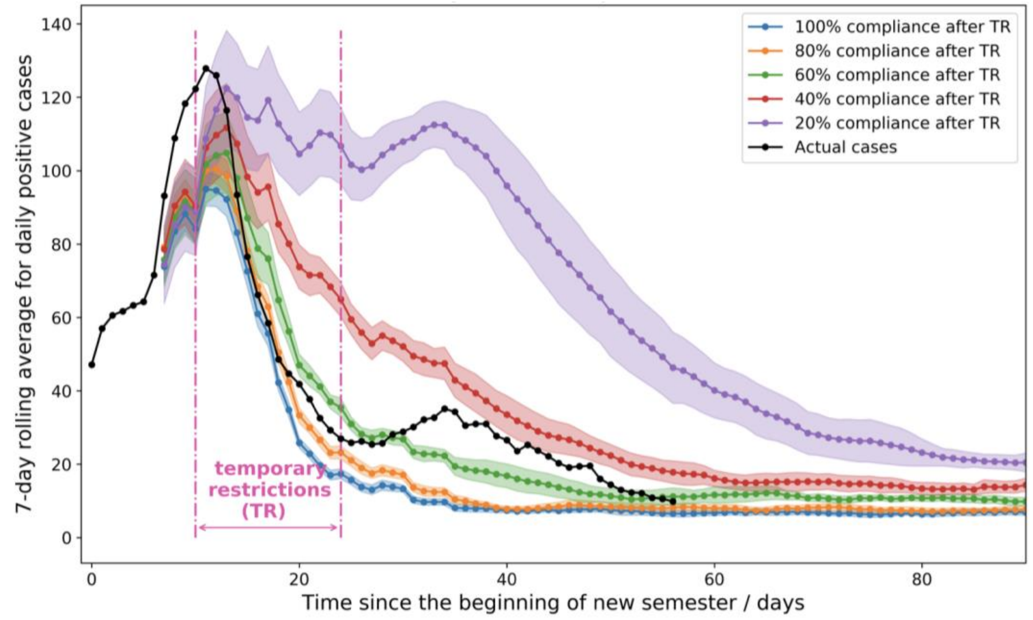
100% compliance after TR
80% compliance after TR
60% compliance after TR
40% compliance after TR
20% compliance after TR
Actual cases
temporary restrictions (TR)
7-day rolling average for daily positive cases
Time since the beginning of new semester / days

c

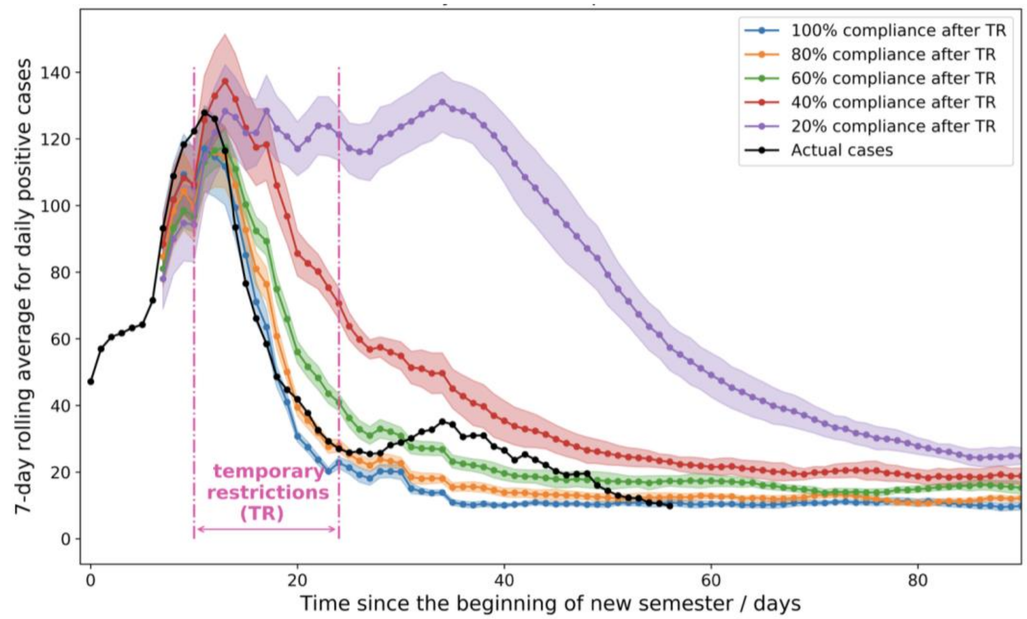
100% compliance after TR
80% compliance after TR
60% compliance after TR
40% compliance after TR
20% compliance after TR
Actual cases
temporary restrictions (TR)
7-day rolling average for daily positive cases
Time since the beginning of new semester / days

Supplementary Fig. 8. 7-day rolling average for daily positive cases as computed using the agent-based model assuming different levels of compliance with isolation and quarantine compared to the confirmed cases detected using covidShield test as of October 15, 2020. (a) Trajectory of cases assuming an average rate of imported infections = 2 cases/day, (b) Trajectory of cases assuming an average rate of imported infections = 6 cases/day, and (c) Trajectory of cases assuming an average rate of imported infections = 10 cases/day. Compliance of the cohort of agents with high social activity levels (e.g., those who attend parties and go to bars) was assumed to be 0% prior to the temporary restrictions (essential activities) period. During the essential activity period, simulated agents are allowed to go to classes, restaurants, libraries, and coffee shops but not to attend bars and parties. Imported infections are introduced to account for incidental infections in the university population due to interaction with the surrounding community which has its own prevalence. The results suggest that the large increase in cases in the beginning of the semester may be explained as a combination of lack of compliance with isolation and quarantine and high levels of imported infections (> 10 cases/day, Panel c). The essential activities period helped contain the spread and led to rapid decline in cases. The model estimates that student compliance during this period was relatively high ranging between 60%-80% (2 daily imported infections, Panel a), 80% (6 daily imported infections, Panel b), and close to 100% (10 daily imported infections, Panel c). After the temporary restrictions ended, the inferred compliance levels, required to match the observed cases, varied but was estimated to be between 40% and 60% with the lower end more probable if imported infections = 2 cases/day and the upper end is more probable if imported infections = 10 cases/day. Between 08/15/2020 and 10/01/2020, and assuming only 25%-50% of covid cases were detected and reported at the state level, the estimated 7-day prevalence in the state of Illinois ranged between 0.22% and 0.33%. For the socially active agent population (approximately 15,000 agents), this corresponds to imported infections ranging between 4.7 cases/day and 9 cases/day consistent with the estimates in Panels b and c. These results suggest that compliance levels with isolation and quarantine were close to 60%, the essential activities period was successful in quickly limiting the spread and higher level of compliance during that period further assisted with this, and community transmission as measured by imported infection is an important confounder in shaping the epidemic curve.

a

b

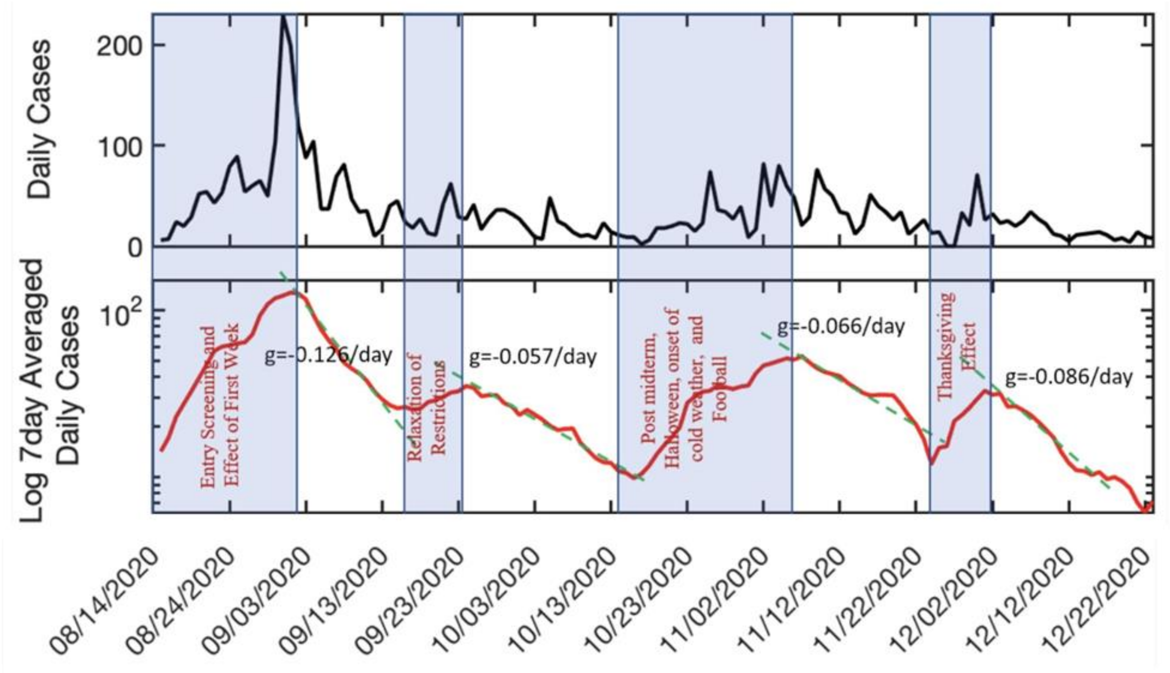

Supplementary Fig. 9. Estimating the effective exponential decay rate that was achieved by Shield during different intervals of Fall 2021. (**a**) Daily new cases by date of sample collection. (**b**) The natural logarithm of the 7-day averaged daily new cases. The slope of the dashed line *g* measures the effective exponential decay rate in the intervals over which cases were decreasing. The exponentail decary rate ranged between g = -0.126/day (halving time = 5.5.days) to g = -0.057/day (halving time = 12.1 days). Assuming a serial time interval that is gamma distributed with a mean = 4 days and standard deviation = 3.25 days, these decay rates correspond to an effective reproduction number[48] that ranges between 0.55 and 0.78. These estimates are consistent with the full time-dependent trajectories displayed in Figure 3 in the main text which demonstrate that R_t reached as low as 0.5, and was frequently around 0.75-0.85.

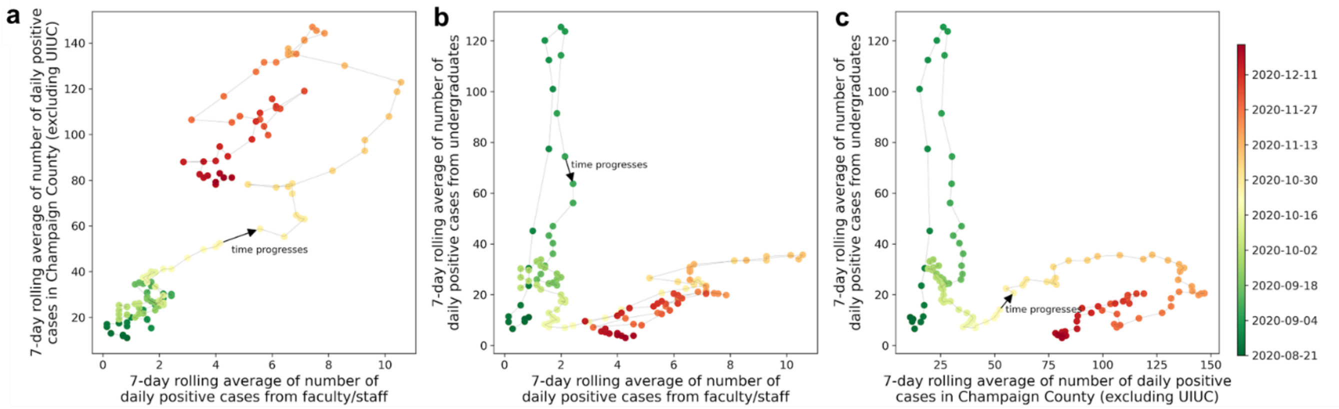

Supplementary Fig. 10. Measured correlations of the number of 7-day averaged daily new cases between residents in Champaign County, faculty/staff, and undergraduate students for the period between August 15 and December 23. All points in three scatter plots are colored according to their dates, as shown in the color bar on the right. (**a**) The number of 7-day averaged daily new cases of residents in Champaign county strongly correlated with that of faculty/staff especially for the period after October 18 (Pearson correlation coefficient 0.86, p-value 7.99×10^{-38} calculated using a two-tailed test). (**b**) At the beginning of the semester (around August 31) when there was a spike in daily new cases from undergraduate students, it had little influence on the faculty/staff. While at the late period of the semester (after October 18) when the case positivity in Illinois increased, they showed a correlation (Pearson correlation coefficient 0.88, p-value 1.33×10^{-21} calculated using a two-tailed test). (**c**) The initial spike in the number of daily new cases of undergraduate students doesn't correlate with that of residents in Champaign county. While at the late period of the semester (after October 18), as the number of positive cases in Champaign county increased and then decreased, the number of cases of undergraduate students also showed a similar trend.

Supplementary Table 1. Comparison of Ct values from 25 clinical samples that were split into two aliquots upon receipt, one set was processed using our covidSHIELD assay and the other set was subjected to RNA extraction using MagMax Viral/Pathogen II (MVP II) Nucleic Acid Isolation Kit (ThermoFisher).

Sample #	Direct saliva Call	RNA extract Call	Direct saliva Ct Values				RNA extract Ct Values			
			ORF1ab	*N*-gene	*S*-gene	MS2	*ORF1ab*	*N*-gene	*S*-gene	MS2
1	POSITIVE	POSITIVE	34.87689	34.7379	35.15069	31.43486	32.53683	33.62223	33.32546	29.20287
2	POSITIVE	POSITIVE	35.77484	31.63383	34.52456	27.39116	31.82251	31.47362	33.69433	24.40539
3	NEGATIVE	NEGATIVE	Undetermined	Undetermined	Undetermined	31.98769	Undetermined	Undetermined	Undetermined	25.35025
4	POSITIVE	POSITIVE	21.35963	19.91812	21.34096	Undetermined	21.15125	20.97386	20.84411	25.25592
5	POSITIVE	POSITIVE	29.7138	28.12146	29.12307	36.42744	28.12993	28.94287	26.80732	23.59188
6	POSITIVE	POSITIVE	31.75227	31.18219	32.32317	30.34586	29.79028	30.34225	29.37346	23.5
7	POSITIVE	POSITIVE	27.67761	26.04979	27.44747	33.67909	24.12692	24.64138	23.57869	22.80227
8	POSITIVE	POSITIVE	36.4927	36.48437	36.93896	28.00187	Undetermined	34.4855	36.89221	22.77695
9	POSITIVE	POSITIVE	18.32149	16.63957	17.65472	Undetermined	14.88425	14.77061	15.18406	26.68707
10	POSITIVE	POSITIVE	30.50895	30.42821	31.71363	35.54698	28.93207	28.85836	29.24729	23.41111
11	POSITIVE	POSITIVE	32.64179	34.5071	36.60606	38.88396	32.00633	32.99638	29.73417	22.02418
12	NEGATIVE	NEGATIVE	Undetermined	Undetermined	Undetermined	30.05719	Undetermined	Undetermined	Undetermined	26.15957
13	NEGATIVE	NEGATIVE	Undetermined	Undetermined	Undetermined	30.69856	Undetermined	Undetermined	Undetermined	25.32277
14	POSITIVE	POSITIVE	24.31471	23.53031	24.30666	29.79528	23.33266	23.90056	23.92258	27.70757
15	POSITIVE	POSITIVE	18.86967	17.92603	19.15773	Undetermined	19.11883	19.35747	19.49679	25.0189
16	POSITIVE	POSITIVE	Undetermined	36.27135	34.65929	30.30633	Undetermined	34.78008	33.06602	24.60403
17	POSITIVE	POSITIVE	26.1404	23.63066	25.59911	31.9001	22.4836	22.44151	22.57868	23.68353
18	POSITIVE	POSITIVE	24.88006	24.41358	25.35321	Undetermined	24.94342	25.0757	25.15791	25.10076
19	POSITIVE	POSITIVE	34.05909	32.84715	33.15377	29.26073	30.61996	32.79143	30.9592	23.24416
20	NEGATIVE	NEGATIVE	31.63252	31.42906	33.89475	32.67826	34.07472	33.28623	35.13592	26.34162
21	POSITIVE	POSITIVE	Undetermined	Undetermined	Undetermined	29.93763	Undetermined	Undetermined	Undetermined	28.28117
22	POSITIVE	POSITIVE	33.39564	31.59387	33.96499	28.2531	28.93732	29.36552	29.37632	25.81484
23	POSITIVE	POSITIVE	17.52907	15.91184	17.7005	Undetermined	14.79202	14.88865	14.93889	31.47698
24	POSITIVE	POSITIVE	32.60616	29.36388	32.66383	30.71958	30.41657	31.16113	30.37413	26.24533
25	POSITIVE	POSITIVE	34.66038	32.72861	34.80508	30.83342	30.00526	31.25149	30.34891	24.93401
Average Ct for viral target genes			28.78				27.14			

Supplementary Table 2. Comparison of method of loading to the 384-well PCR plate (manual vs. multichannel robot) and the method of loading heat-inactivated saliva samples to the 96-deep-well plates pre-loaded with 2xTBE/1% Tween-20 buffer (manual vs. Span8 robot).

SARS-CoV-2 viral copies/mL	Sample #	saliva in 50mL tube to 96-deep-well plate	96-deep-well to 384-well PCR plate	qPCR machine	Ct values				Call
					ORF1ab	*N*-gene	*S*-gene	MS2	
500	1	manual	multichannel robot	QS7Pro	33.9	34.9	34.3	30.2	POSITIVE
500	2	manual	multichannel robot	QS7Pro	36.3	33.7	34.2	27.8	POSITIVE
500	3	manual	multichannel robot	QS7Pro	35.9	33.7	33.7	27.9	POSITIVE
500	4	manual	multichannel robot	QS7Pro	35.3	33.7	34.5	28.8	POSITIVE
500	5	manual	multichannel robot	QS7Pro	35.7	33.1	33.6	27.9	POSITIVE
500	6	manual	multichannel robot	QS7Pro	35.9	33.4	Undetermined	26.8	POSITIVE
500	7	manual	multichannel robot	QS7Pro	Undetermined	33.3	33.9	27.8	POSITIVE
500	8	manual	multichannel robot	QS7Pro	35.0	34.1	35.2	27.0	POSITIVE
500	9	manual	multichannel robot	QS7Pro	Undetermined	35.0	36.4	31.0	POSITIVE
500	10	manual	multichannel robot	QS7Pro	33.6	33.4	33.4	28.9	POSITIVE
500	11	manual	multichannel robot	QS7Pro	34.1	33.3	36.3	27.8	POSITIVE
500	12	manual	multichannel robot	QS7Pro	35.0	34.7	37.1	27.8	POSITIVE
500	13	manual	multichannel robot	QS7Pro	34.0	34.0	37.0	27.9	POSITIVE
500	14	manual	multichannel robot	QS7Pro	33.9	33.1	34.6	27.6	POSITIVE
500	15	manual	multichannel robot	QS7Pro	36.3	34.4	Undetermined	27.3	POSITIVE
500	16	manual	multichannel robot	QS7Pro	36.5	34.4	35.3	27.7	POSITIVE
500	17	manual	multichannel robot	QS7Pro	33.9	34.1	36.9	28.1	POSITIVE
500	18	manual	multichannel robot	QS7Pro	34.0	35.0	34.7	27.7	POSITIVE
500	19	manual	multichannel robot	QS7Pro	36.0	34.8	37.1	28.1	POSITIVE
500	20	manual	multichannel robot	QS7Pro	34.9	34.2	34.4	27.8	POSITIVE
500	1	manual	manual	QS7Pro	34.9	34.3	36.2	28.7	POSITIVE
500	2	manual	manual	QS7Pro	35.8	35.1	34.6	30.0	POSITIVE
500	3	manual	manual	QS7Pro	33.6	34.1	34.1	27.5	POSITIVE
500	4	manual	manual	QS7Pro	Undetermined	33.6	33.6	29.0	POSITIVE
500	5	manual	manual	QS7Pro	33.5	32.9	35.2	28.4	POSITIVE

500	6	manual	manual	QS7Pro	34.6	33.6	35.2	27.8	POSITIVE
500	7	manual	manual	QS7Pro	37.0	34.3	34.1	29.0	POSITIVE
500	8	manual	manual	QS7Pro	34.0	33.3	36.1	27.5	POSITIVE
500	9	manual	manual	QS7Pro	34.3	32.6	34.5	27.6	POSITIVE
500	10	manual	manual	QS7Pro	36.6	36.6	35.1	27.5	POSITIVE
500	11	manual	manual	QS7Pro	34.7	33.4	34.7	28.1	POSITIVE
500	12	manual	manual	QS7Pro	34.3	33.7	32.8	28.1	POSITIVE
500	13	manual	manual	QS7Pro	35.0	33.9	35.3	28.8	POSITIVE
500	14	manual	manual	QS7Pro	33.5	33.1	33.8	28.5	POSITIVE
500	15	manual	manual	QS7Pro	34.5	34.3	35.5	28.3	POSITIVE
500	16	manual	manual	QS7Pro	33.7	33.8	34.4	27.6	POSITIVE
500	17	manual	manual	QS7Pro	35.2	33.7	34.1	27.4	POSITIVE
500	18	manual	manual	QS7Pro	35.6	33.7	38.2	28.4	POSITIVE
500	19	manual	manual	QS7Pro	33.8	33.7	34.1	27.3	POSITIVE
500	20	manual	manual	QS7Pro	33.0	33.7	33.8	27.4	POSITIVE
500	1	Span8 robot	multichannel robot	QS7Pro	35.2	34.4	Undetermined	26.6	POSITIVE
500	2	Span8 robot	multichannel robot	QS7Pro	34.9	34.9	39.7	26.2	POSITIVE
500	3	Span8 robot	multichannel robot	QS7Pro	35.3	34.6	35.8	26.0	POSITIVE
500	4	Span8 robot	multichannel robot	QS7Pro	36.6	34.9	35.8	25.5	POSITIVE
500	5	Span8 robot	multichannel robot	QS7Pro	37.7	34.1	35.3	25.8	POSITIVE
500	6	Span8 robot	multichannel robot	QS7Pro	34.7	34.3	38.9	25.8	POSITIVE
500	7	Span8 robot	multichannel robot	QS7Pro	39.5	35.6	36.3	25.8	POSITIVE
500	8	Span8 robot	multichannel robot	QS7Pro	34.6	36.9	34.4	25.8	POSITIVE
500	9	Span8 robot	multichannel robot	QS7Pro	38.0	34.2	35.9	25.9	POSITIVE
500	10	Span8 robot	multichannel robot	QS7Pro	34.3	34.4	39.1	26.1	POSITIVE
500	11	Span8 robot	multichannel robot	QS7Pro	33.7	33.8	37.0	26.3	POSITIVE
500	12	Span8 robot	multichannel robot	QS7Pro	Undetermined	35.3	35.3	26.0	POSITIVE
500	13	Span8 robot	multichannel robot	QS7Pro	34.5	34.4	35.4	26.2	POSITIVE
500	14	Span8 robot	multichannel robot	QS7Pro	36.6	34.1	34.5	26.0	POSITIVE
500	15	Span8 robot	multichannel robot	QS7Pro	37.5	35.3	34.7	26.1	POSITIVE
500	16	Span8 robot	multichannel robot	QS7Pro	35.0	33.4	39.2	26.1	POSITIVE
500	17	Span8 robot	multichannel robot	QS7Pro	35.1	35.1	33.8	26.0	POSITIVE
500	18	Span8 robot	multichannel robot	QS7Pro	34.1	33.6	33.7	26.0	POSITIVE
500	19	Span8 robot	multichannel robot	QS7Pro	34.6	34.7	39.3	26.0	POSITIVE

500	20	Span8 robot	multichannel robot	QS7Pro	33.2	33.8	35.4	26.1	POSITIVE
					ORF1ab	*N*-gene	*S*-gene		
		Average manual → robot			35.0	34.0	35.1		
		Average manual →manual			34.6	33.9	34.8		
		Average robot → robot			35.5	34.6	36.3		

We compared the method of loading samples to the 96-deep-well plate (manual vs. Span8 robot) and the method of loading to the 384-well PCR plate (manual vs. multichannel robot) as variables in a linear model. No significant difference was observed, with a calculated respective *p*-value of p=0.127 and p=0.24, calculated using 2-tailed, unpaired t-test. We also completed an ANOVA of the different combinations used, with Tukey's Honestly Significant Difference to look at contrasts, and found that there were no significant differences overall (p=0.277) or in any pairs compared: (1) manual load to 96-deep well plate and manual load to 384-well PCR plate vs. manual load to 96-deep well plate and multichannel robot to 384-well PCR plate p=0.465; (2) manual load to 96-deep well plate and manual load to 384-well PCR plate vs. Span8 robot to 96-deep well plate and multichannel robot to 384-well PCR plate p=0.931; and (3) Span8 robot to 96-deep well plate and multichannel robot to 384-well PCR plate *vs*. manual load to 96-deep well plate and multichannel robot to 384-well PCR plate p=0.277.

Supplementary Table 3. Endogenous & Exogenous Interference testing results for covidSHIELD assay

Potential Interfering Substances	Concentration	Negative Samples	Positive Samples
		Detected	Detected
Saliva Samples			
Nasal congestion spray	15% v/v	3/3 Negative	3/3 Positive
NeilMed Nasogel	1.25%	3/3 Negative	3/3 Positive
Cepacol Lozenges (benzocaine/menthol)	3 mg/mL	3/3 Negative	3/3 Positive
Chloroseptic Sore Throat spray	5% v/v	3/3 Negative	3/3 Positive
Crest/Listerine Mouthwash	5% v/v	3/3 Negative	3/3 Positive
Act dry mouth lozenges	3 mg/mL	3/3 Negative	3/3 Positive
Toothpaste (Colgate)	0.5% v/v	3/3 Negative	1/3 Negative<br>2/3 Positive
Mucin: bovine submaxillary gland, type I-S	2.5 mg/ml	3/3 Negative	3/3 Positive
Human Genomic DNA	10 ng/µl	3/3 Negative	3/3 Positive
White blood cells/Leukocytes	1 to 5x10^6 cells/mL	3/3 Negative	3/3 Positive
Nicotine	0.03 mg/mL	3/3 Negative	3/3 Positive

Supplementary Table 4. Qualitative outcome of parallel testing of paired mid-turbinate swabs and saliva with the Abbott RealTime SARS-CoV-2 assay and covidSHIELD.

ID #		Comparator type	Comparator Result (POSITIVE or NEGATIVE)	Comparator CN Value	covidSHIELD Result (POSITIVE or NEGATIVE)	covidSHIELD *S*-gene CT Value	covidSHIELD *N*-gene CT Value	covidSHIELD *ORF1ab* gene CT Value	Comments/ Notes	Retest covidSHIELD *S*-gene CT Value	Retest covidSHIELD *N*-gene CT Value	Retest covidSHIELD *ORF1ab* gene CT Value
1		NP Swab	POSITIVE	13.52	POSITIVE	22.81	21.97	22.08				
2		NP Swab	NEGATIVE	-1	NEGATIVE	Undetermined	Undetermined	Undetermined				
3		NP Swab	POSITIVE	6.03	POSITIVE	24.77	23.6	24.3				
4		NP Swab	NEGATIVE	-1	NEGATIVE	Undetermined	Undetermined	Undetermined				
5		NP Swab	NEGATIVE	-1	NEGATIVE	Undetermined	Undetermined	Undetermined				
6		NP Swab	POSITIVE	19.82	POSITIVE	Undetermined	35.57	Undetermined	Saliva sample was inconclusive with only 1 viral gene Ct<39. Per protocol, sample was rerun and result was positive.	36.68	36.13	35.90
7		NP Swab	POSITIVE	6.54	POSITIVE	35.62	33.45	38.4				
8		NP Swab	NEGATIVE	-1	NEGATIVE	Undetermined	Undetermined	Undetermined				
9		NP Swab	NEGATIVE	-1	NEGATIVE	Undetermined	Undetermined	Undetermined				
10		NP Swab	NEGATIVE	-1	POSITIVE	36.29	35.96	35.38				
11		NP Swab	NEGATIVE	-1	NEGATIVE	Undetermined	Undetermined	Undetermined				
12		NP Swab	NEGATIVE	-1	NEGATIVE	Undetermined	Undetermined	Undetermined				
13		NP Swab	NEGATIVE	-1	NEGATIVE	Undetermined	Undetermined	Undetermined				
14		NP Swab	NEGATIVE	-1	NEGATIVE	Undetermined	Undetermined	Undetermined				
15		NP Swab	NEGATIVE	-1	NEGATIVE	Undetermined	Undetermined	Undetermined				
16		NP Swab	POSITIVE	10.95	POSITIVE	29.29	27.01	28.42				
17		NP Swab	POSITIVE	7.56	POSITIVE	26.74	25.32	26.89				
18		NP Swab	NEGATIVE	-1	NEGATIVE	Undetermined	Undetermined	Undetermined				
19		NP Swab	POSITIVE	14.58	POSITIVE	33.95	30.98	32.57				
20		NP Swab	NEGATIVE	-1	NEGATIVE	Undetermined	Undetermined	Undetermined				
21		NP Swab	NEGATIVE	-1	NEGATIVE	Undetermined	Undetermined	Undetermined				
22		NP Swab	NEGATIVE	-1	NEGATIVE	Undetermined	Undetermined	Undetermined				
23		NP Swab	NEGATIVE	-1	NEGATIVE	Undetermined	Undetermined	Undetermined				
24		NP Swab	NEGATIVE	-1	NEGATIVE	Undetermined	Undetermined	Undetermined				
25		NP Swab	POSITIVE	14.58	POSITIVE	36.43	32.46	34.31				
26		NP Swab	POSITIVE	22.01	POSITIVE	28.84	26.7	28.39				
27		NP Swab	NEGATIVE	-1	NEGATIVE	Undetermined	Undetermined	Undetermined				
28		NP Swab	NEGATIVE	-1	NEGATIVE	Undetermined	Undetermined	Undetermined				
29		NP Swab	POSITIVE	24.61	NEGATIVE	Undetermined	Undetermined	Undetermined				
30		NP Swab	NEGATIVE	-1	NEGATIVE	Undetermined	Undetermined	Undetermined				
31		NP Swab	NEGATIVE	-1	NEGATIVE	Undetermined	Undetermined	Undetermined				
32		NP Swab	POSITIVE	11.22	POSITIVE	23.77	21.96	23.15				

33		NP Swab	NEGATIVE	-1	NEGATIVE	Undetermined	Undetermined	Undetermined				
34		NP Swab	POSITIVE	14.97	POSITIVE	24.6	23.98	24.45				
35		NP Swab	POSITIVE	7.81	POSITIVE	29.92	29.3	29.71				
36		NP Swab	POSITIVE	11.26	POSITIVE	27.65	26.86	27.73				
37		NP Swab	POSITIVE	16.14	POSITIVE	29.5	28.83	30.15				
38		NP Swab	POSITIVE	17.72	POSITIVE	25.93	24.27	26.32				
39		NP Swab	POSITIVE	6.09	POSITIVE	26.64	25.3	26.26				
40		NP Swab	POSITIVE	16.24	POSITIVE	28.07	26.39	27.67				
41		NP Swab	POSITIVE	8.84	POSITIVE	28.79	26.17	27.59				
42		NP Swab	POSITIVE	7.95	POSITIVE	35	32.8	34.9				
43		NP Swab	POSITIVE	4.6	POSITIVE	27.21	25.77	27.02				
44		NP Swab	NEGATIVE	-1	NEGATIVE	Undetermined	Undetermined	Undetermined				
45		NP Swab	NEGATIVE	-1	NEGATIVE	Undetermined	Undetermined	Undetermined				
46		NP Swab	NEGATIVE	-1	NEGATIVE	Undetermined	Undetermined	Undetermined				
47		NP Swab	NEGATIVE	-1	NEGATIVE	Undetermined	Undetermined	Undetermined				
48		NP Swab	NEGATIVE	-1	NEGATIVE	Undetermined	Undetermined	Undetermined				
49		NP Swab	NEGATIVE	-1	NEGATIVE	Undetermined	Undetermined	Undetermined				
50		NP Swab	POSITIVE	10.24	POSITIVE	26.25	25.27	26.68				
51		NP Swab	POSITIVE	8.58	POSITIVE	28.03	26.44	27.72				
52		NP Swab	NEGATIVE	-1	NEGATIVE	Undetermined	Undetermined	Undetermined				
53		NP Swab	NEGATIVE	-1	NEGATIVE	Undetermined	Undetermined	Undetermined				
54		NP Swab	NEGATIVE	-1	NEGATIVE	Undetermined	Undetermined	Undetermined				
55		NP Swab	NEGATIVE	-1	NEGATIVE	Undetermined	Undetermined	Undetermined				
56		NP Swab	NEGATIVE	-1	NEGATIVE	Undetermined	Undetermined	Undetermined				
57		NP Swab	NEGATIVE	-1	NEGATIVE	Undetermined	Undetermined	Undetermined				
58		NP Swab	NEGATIVE	-1	NEGATIVE	Undetermined	Undetermined	Undetermined				
59		NP Swab	NEGATIVE	-1	NEGATIVE	Undetermined	Undetermined	Undetermined				
60		NP Swab	NEGATIVE	-1	NEGATIVE	Undetermined	Undetermined	Undetermined				
61		NP Swab	NEGATIVE	-1	NEGATIVE	Undetermined	Undetermined	Undetermined				
62		NP Swab	NEGATIVE	-1	NEGATIVE	Undetermined	Undetermined	Undetermined				
63		NP Swab	NEGATIVE	-1	NEGATIVE	Undetermined	Undetermined	Undetermined				
64		NP Swab	NEGATIVE	-1	NEGATIVE	Undetermined	Undetermined	Undetermined				
65		NP Swab	POSITIVE	7.18	POSITIVE	25.01	24.39	24.97				
66		NP Swab	POSITIVE	7.36	POSITIVE	22.35	21.79	22.28				
67		MT Swab	POSITIVE	8.01	POSITIVE	33.06	30.76	32.11				
68		MT Swab	POSITIVE	17.81	POSITIVE	28.47	26.97	27.46				
69		MT Swab	POSITIVE	5.1	POSITIVE	25.06	24.05	24.70				
70		MT Swab	POSITIVE	3.29	POSITIVE	29.63	28.1	28.85				
71		MT Swab	POSITIVE	11.62	POSITIVE	36.72	37.36	Undetermined				
72		MT Swab	NEGATIVE	-1	NEGATIVE	Undetermined	Undetermined	Undetermined				
73		MT Swab	NEGATIVE	-1	NEGATIVE	Undetermined	Undetermined	Undetermined				
74		MT Swab	NEGATIVE	-1	NEGATIVE	Undetermined	Undetermined	Undetermined				
75		MT Swab	NEGATIVE	-1	NEGATIVE	Undetermined	Undetermined	Undetermined				

76		MT Swab	NEGATIVE	-1	NEGATIVE	Undetermined	Undetermined	Undetermined				
77		MT Swab	NEGATIVE	-1	NEGATIVE	Undetermined	Undetermined	Undetermined				
78		MT Swab	NEGATIVE	-1	NEGATIVE	Undetermined	Undetermined	Undetermined				
79		MT Swab	NEGATIVE	-1	NEGATIVE	Undetermined	Undetermined	Undetermined				
80		MT Swab	NEGATIVE	-1	NEGATIVE	Undetermined	Undetermined	Undetermined				
81		MT Swab	NEGATIVE	-1	NEGATIVE	Undetermined	Undetermined	Undetermined				
82		MT Swab	NEGATIVE	-1	NEGATIVE	Undetermined	Undetermined	Undetermined				
83		MT Swab	NEGATIVE	-1	NEGATIVE	Undetermined	Undetermined	Undetermined				
84		MT Swab	NEGATIVE	-1	NEGATIVE	Undetermined	Undetermined	Undetermined				
85		MT Swab	NEGATIVE	-1	NEGATIVE	Undetermined	Undetermined	Undetermined				
86		MT Swab	NEGATIVE	-1	NEGATIVE	Undetermined	Undetermined	Undetermined				
87		MT Swab	NEGATIVE	-1	NEGATIVE	Undetermined	Undetermined	Undetermined				
88		MT Swab	NEGATIVE	-1	NEGATIVE	Undetermined	Undetermined	Undetermined				
89		MT Swab	NEGATIVE	-1	NEGATIVE	Undetermined	Undetermined	Undetermined				
90		MT Swab	NEGATIVE	-1	NEGATIVE	Undetermined	Undetermined	Undetermined				
91		MT Swab	NEGATIVE	-1	NEGATIVE	Undetermined	Undetermined	Undetermined				
92		MT Swab	NEGATIVE	-1	NEGATIVE	Undetermined	Undetermined	Undetermined				
93		MT Swab	NEGATIVE	-1	NEGATIVE	Undetermined	Undetermined	Undetermined				
94		MT Swab	NEGATIVE	-1	NEGATIVE	Undetermined	Undetermined	Undetermined				
95		MT Swab	NEGATIVE	-1	NEGATIVE	Undetermined	Undetermined	Undetermined				
96		MT Swab	NEGATIVE	-1	NEGATIVE	Undetermined	Undetermined	Undetermined				
97		MT Swab	NEGATIVE	-1	NEGATIVE	Undetermined	Undetermined	Undetermined				
98		MT Swab	NEGATIVE	-1	NEGATIVE	Undetermined	Undetermined	Undetermined				
99		MT Swab	NEGATIVE	-1	NEGATIVE	Undetermined	Undetermined	Undetermined				
100		MT Swab	NEGATIVE	-1	NEGATIVE	Undetermined	Undetermined	Undetermined				
101		MT Swab	POSITIVE	3.5	POSITIVE	19.92	19.14	19.82	Nasal test was at first inconclusive, then when rerun, was inconclusive. Path lab created a 1:3 dilution, which then resulted in positive result, presented here. The path lab director wrote "The assay was giving errors because that viral load is above the upper limit of quantification".			
102		MT Swab	NEGATIVE	-1	NEGATIVE	Undetermined	Undetermined	Undetermined				
103		MT Swab	NEGATIVE	-1	NEGATIVE	Undetermined	Undetermined	Undetermined				
104		MT Swab	NEGATIVE	-1	NEGATIVE	Undetermined	Undetermined	Undetermined				

105		MT Swab	NEGATIVE	-1	NEGATIVE	Undetermined	Undetermined	Undetermined				
106		MT Swab	NEGATIVE	-1	NEGATIVE	Undetermined	Undetermined	Undetermined				
107		MT Swab	NEGATIVE	-1	NEGATIVE	Undetermined	Undetermined	Undetermined				
108		MT Swab	NEGATIVE	-1	NEGATIVE	Undetermined	Undetermined	Undetermined				
109		MT Swab	NEGATIVE	-1	NEGATIVE	Undetermined	Undetermined	Undetermined				
110		MT Swab	NEGATIVE	-1	NEGATIVE	Undetermined	Undetermined	Undetermined				
111		MT Swab	NEGATIVE	-1	NEGATIVE	Undetermined	Undetermined	Undetermined				
112		MT Swab	NEGATIVE	-1	NEGATIVE	Undetermined	Undetermined	Undetermined				
113		MT Swab	NEGATIVE	-1	NEGATIVE	Undetermined	Undetermined	Undetermined				
114		MT Swab	NEGATIVE	-1	NEGATIVE	Undetermined	Undetermined	Undetermined				
115		MT Swab	NEGATIVE	-1	NEGATIVE	Undetermined	Undetermined	Undetermined				
116		MT Swab	NEGATIVE	-1	NEGATIVE	Undetermined	Undetermined	Undetermined				
117		MT Swab	NEGATIVE	-1	NEGATIVE	Undetermined	Undetermined	Undetermined				
118		MT Swab	NEGATIVE	-1	NEGATIVE	Undetermined	Undetermined	Undetermined				
119		MT Swab	NEGATIVE	-1	NEGATIVE	Undetermined	Undetermined	Undetermined				
120		MT Swab	NEGATIVE	-1	NEGATIVE	Undetermined	Undetermined	Undetermined				

Supplementary Table 5. Method comparison study completed to support the correlation between saliva samples processed with covidSHIELD and nasal samples processed with Abbott RealTime SARS-CoV-2 assay performed on the Abbott m2000 System.

Name of Candidate device	covidSHIELD
Study Title	Method comparison study for covidSHIELD to an RT-qPCR nasopharyngeal or mid-turbinate reference method for detection of SARS-CoV-2.
Study Type (CLIA – high/moderate complexity, CLIA waived/POC, OTC, usability, other)	The candidate device is a high complexity assay only housed in high complexity CLIA-certified laboratories<br><br>Saliva samples were self-collected with observation
Number of Study Sites	Samples were collected at 4 different sites (one in Urbana, IL; One in Madison, WI; two in Chicago, IL); candidate testing was done at one testing site.
Operators	Operators processed samples for comparator and candidate in high complexity labs with appropriate trained personnel.
Target Population	Study population included:<br><br>Participants over the age of 18<br>Participants suspected of COVID-19 (either with symptoms or with known exposures to someone positive for COVID-19).
Number of Participants	We enrolled a total of 120 participants. We had 31 positives and 89 negatives as determined by the comparator (either an MT or NP swab, as available at each site).
Study Objective	To determine the positive percent agreement (PPA) and negative percent agreement (NPA) between the candidate device on saliva samples and an EUA-authorized RT-qPCR assay (specifically, the Abbott RealTime SARS-CoV-2 assay with an LoD of 2,700 NDU/ml according to the list of FDA Reference Panel assays).
Study Comparator	Abbott RealTime SARS-CoV-2 assay performed on the Abbott m2000 System

Comparator Device Sample matrix and collection method	The comparator sample was either a mid-turbinate or nasopharyngeal swab collected by a healthcare professional. Each swab was placed into a vial of collection media and transported to the test instrument (per the comparator manufacturer's instructions).
Study General Design and Duration	Participants were approached:<br>• Prospectively while visiting a saliva collection site (Urbana) OR<br>• Prospectively while visiting one of two different urgent care centers (Chicago) OR<br>• Following a recent anterior nares swab positive test for SARS-CoV-2. Approximately 24 hours following the anterior nares swab collection, those individuals who tested positive were asked to consent to a research study. (Madison)<br><br>After consent, participants answered survey questions about symptoms and provided a self-collected saliva specimen and either a mid-turbinate or nasopharyngeal swab specimen (as available at each site), collected by a healthcare professional.
Participant & Sample Inclusion / Exclusion Criteria	Inclusion Criteria for Participants:<br>Participants meeting the following criteria were included in this study:<br><br>1. Participant is 18 years of age or older<br>2. Participant must be willing and able to provide a saliva sample and willing and able to have a healthcare professional successfully collect a mid-turbinate or nasopharyngeal sample<br>3. Participant must be willing and able to answer questions about their symptoms as part of the data collected for this study<br>4. Participant self-reported being symptomatic<br><br>Exclusion Criteria for Participants:<br>Patients with the following criteria were excluded:<br><br>1. Participants under 18 years of age<br>2. Participants who have previously tested positive for SARS-CoV-2, indicating that they have previously been infected with SARS-CoV-2.

	3. Participants who have had anything "by mouth" (eaten, drank, brushed their teeth, smoked or chewed gum etc.) in the last 60 minutes.<br><br>Exclusion Criteria for Samples:<br><br>1. If sample tube label was illegible, the sample would have been excluded from the study<br>2. If sample tube had evidence of leaking or volume loss, the sample would have been excluded from the study<br>3. If sample had visual debris present or any obvious contamination due to blood, food or beverage etc, the sample would have been excluded from the study.
Study Procedures	• Participants were instructed to provide a saliva sample by allowing saliva to collect in the mouth and gently expelling saliva into the collection tube. Sample donors then capped their tube. The participant handed the sample to the healthcare professional or collection site staff who placed the tube in a collection container.<br>• The participant was asked survey questions about their symptoms.<br>• The healthcare professional collected the mid-turbinate or nasopharyngeal sample and transferred the swab into the collection media (per the comparator manufacturer's instructions).<br>• Both samples were transported (via ground) to the testing location. All saliva samples were tested at the UIUC CLIA-certified lab on the candidate test; all swabs were transported by ground to the University of Illinois Chicago Hospital where they were tested with the Abbott RealTime SARS-CoV-2 assay performed on the Abbott m2000 System.
Statistical Analysis	• Positive percent agreement (PPA) and negative percent agreement (NPA) were determined by comparing results from the candidate test with those from the comparator test.

Supplementary Table 6. Details to Capture on Case Report Form

Testing of External Controls	Candidate Assay:<br><br>For every test run performed, a known positive and negative control was included in the covidSHELD test's reaction plate, as well as internal controls for each sample. The negative control consisted of UltraPure DNase- and RNase-free water, in order to evaluate cross-contamination between loading samples into the 384-well reaction plate. The positive control consisted of the Thermo Fisher TaqPath COVID-19 Combo Kit provided positive control, in order to evaluate the RT-qPCR reagents (primers, probes, Master Mix) and the ability of the QuantStudio 4, 7 Flex, and 7 Pro systems to detect viral genes. The internal control consisted of the Thermo Fisher TaqPath COVID-19 Combo Kit provided MS2, which was spiked into the MasterMix reaction, in order to evaluate if material within the saliva interfered with the detection of viral RNA by RT-qPCR. Controls were included on every plate. Beckman Biomek i5 Span8 and Multichannel robotics were used to transfer samples for some of the tests; for other tests, samples were transferred via manual pipetting.<br><br>The test results were evaluated according to the interpretation tables in the EUA Summary (https://www.fda.gov/media/146317/download).<br><br>Comparator Assay:<br>The control protocol was followed according to standard practice in the clinical laboratory and per manufacturer's instructions.
Sample Storage for candidate device	Saliva samples were stored and/or shipped at room temperature for no longer than 7 days in the capped sample collection tube prior to testing.

Supplementary Table 7. Comparison of mid-turbinate (MT) swab and saliva from 17 individuals identified with low viral load based on MT swab analyzed using Abbott Alinity RT-PCR.

Sample #	Mid-turbinate swab		Saliva			
	Alinity Ct	Result	*S*-gene Ct	*N*-gene Ct	*ORF1ab*gene Ct	Result
1	32.83	positive	34.07	33.39	35.19	positive
2	36.41	positive	33.14	31.68	32.72	positive
3	35.91	positive	29.49	28.94	29.7	positive
4	40.13	positive	34.61	32.19	34.71	positive
5	39.97	positive	30.52	29.82	30.59	positive
6	38.63	positive	35.09	33.68	35.27	positive
7	37.45	positive	34.66	33.12	33.94	positive
8	37.04	positive	33.87	32.2	33.26	positive
9	35.66	positive	27.19	25.79	27.38	positive
10	37.68	positive	35.17	34.77	37.61	positive
11	37.74	positive	34.99	33.81	34.65	positive
12	40.47	positive	33.93	33.99	36.53	positive
13	33.36	positive	undetermined	36.26	undetermined	inconclusive
14	32.48	positive	21.75	21.34	21.37	positive
15	32.48	positive	28.17	27.72	28.23	positive
16	41.73	positive	35.95	34.23	undetermined	positive
17	39.83	positive	37.41	39.66	36.55	positive

Supplementary Table 8. Quanta emission rates, and ventilation rates in different zone types. How many zones in each type is given in the "Count" column.

Zone type	Count	E_i ($quanta/hour$)	r_{vent} ($hour^{-1}$)
Bar	20	150	15
Restaurant	200	20	10
Cafe	50	15	10
Library	50	4	3.5
Classroom	790	Professor :100<br>Student:4	3.5
Dorm	300	4	3.5
Dorm parties	300	150	3.5

Supplementary Table 9: Assumption for how long agents stay in different zones.

Zone type	Time Interval	Time Distribution
Home/Dorm	1h to 8h	Uniform
Library	1h to 7h	Uniform
Cafe	0.5h to 4h	Uniform
Bar	2h to 9h	Uniform
Dorm party	2h	exact 2 hours
