# Supplementary material for: Mitigation of SARS-CoV-2 transmission at a large public university
Source: Nat Commun. 2022 Jun 9;13:3207. doi: 10.1038/s41467-022-30833-3 (PMC9184485; doi:10.1038/s41467-022-30833-3)
Supplement: Supplementary file 2 — Reporting Summary [file 41467_2022_30833_MOESM2_ESM.pdf]

nature research

Corresponding author(s): Martin D. Burke
Paul J. Hergenrother
Timothy M. Fan
Nigel D. Goldenfeld
Rebecca L. Smith
William C. Sullivan
Ahmed Elbanna

Last updated by author(s): 04/28/2022

Reporting Summary

Nature Research wishes to improve the reproducibility of the work that we publish. This form provides structure for consistency and transparency in reporting. For further information on Nature Research policies, see our Editorial Policies and the Editorial Policy Checklist.

Statistics

For all statistical analyses, confirm that the following items are present in the figure legend, table legend, main text, or Methods section.

n/a	Confirmed	
☐	☒	The exact sample size (*n*) for each experimental group/condition, given as a discrete number and unit of measurement
☐	☒	A statement on whether measurements were taken from distinct samples or whether the same sample was measured repeatedly
☐	☒	The statistical test(s) used AND whether they are one- or two-sided<br>*Only common tests should be described solely by name; describe more complex techniques in the Methods section.*
☐	☒	A description of all covariates tested
☐	☒	A description of any assumptions or corrections, such as tests of normality and adjustment for multiple comparisons
☐	☒	A full description of the statistical parameters including central tendency (e.g. means) or other basic estimates (e.g. regression coefficient) AND variation (e.g. standard deviation) or associated estimates of uncertainty (e.g. confidence intervals)
☐	☒	For null hypothesis testing, the test statistic (e.g. *F*, *t*, *r*) with confidence intervals, effect sizes, degrees of freedom and *P* value noted<br>*Give P values as exact values whenever suitable.*
☐	☒	For Bayesian analysis, information on the choice of priors and Markov chain Monte Carlo settings
☐	☒	For hierarchical and complex designs, identification of the appropriate level for tests and full reporting of outcomes
☐	☒	Estimates of effect sizes (e.g. Cohen's *d*, Pearson's *r*), indicating how they were calculated

Our web collection on statistics for biologists contains articles on many of the points above.

Software and code

Policy information about availability of computer code

Data collection

Prior to the COVID-19 pandemic, a large collaborative effort led by the Safe, Healthy Community Initiative on campus had been developing an open source software platform called Rokwire.41 Rokwire is designed to make it easy for individuals and organizations to build apps for mobile devices that support smarter, healthier communities. We had been using the University of Illinois at Urbana-Champaign campus as a test bed for the development of Rokwire — the platform — and the first app built upon it — the Illinois app. Safer Illinois was built on the Rokwire platform and the source code was made open source August 14, 2020. We built privacy into Safer Illinois from the ground up. We made modifications to a beta version of the app to minimize the data we collected and stored so that collected only the data necessary to allow the app to function.
We designed the app to store data related to Exposure Notification for the shortest possible period and then delete it. We ensured that users could delete their data at any time from both the app and servers. We made our privacy notice novice friendly, so that all consent language allowed users to understand up front exactly what data we collect, what we do with that data, how long we keep it, and how users can manage their data.

Data Availability

Aggregate case and testing data are publicly available at https://go.illinois.edu/COVIDTestingData

All other data may be requested through the COVID Research Oversight Committee at https://redcap.link/crocdatasamples

Link to the code for analysis of county-level mortality in the BigTen: https://github.com/juel15401/Big10UniversityCounties_COVID.git

https://doi.org/10.5281/zenodo.6481689

Code Availability
Source code repository of "Safer Illinois" App - the official COVID-19 app of the University of Illinois: https://github.com/rokwire/safer-illinois-app (Ref 64)
https://doi.org/10.5281/zenodo.6493203

Data analysis	Data analysis are described in detail under the Methods section. Ct values were calculated using Design & Analysis software v2.4.1. Data were plotted using GraphPad Prism software v9.3.1, Matlab 9.10.0.1684407(R2021a), or Epidemiological toolbox EpiNow2. Statistical tests were computed using either GraphPad Prism software v9.3.1 or Matlab 9.10.0.1684407(R2021a)

For manuscripts utilizing custom algorithms or software that are central to the research but not yet described in published literature, software must be made available to editors and reviewers. We strongly encourage code deposition in a community repository (e.g. GitHub). See the Nature Research guidelines for submitting code & software for further information.

Data

Policy information about availability of data

All manuscripts must include a data availability statement. This statement should provide the following information, where applicable:

- Accession codes, unique identifiers, or web links for publicly available datasets
- A list of figures that have associated raw data
- A description of any restrictions on data availability

Data for clinical studies can be found in Supplementary Tables 1, 4 and 7.
EUA202555 SUMMARY covidSHIELD Assay (https://www.fda.gov/media/146317/download)
Aggregate case and testing data are publicly available at https://go.illinois.edu/COVIDTestingData
All other data may be requested through the COVID Research Oversight Committee at https://forms.illinois.edu/sec/1409755003
Link to the code for analysis of county-level mortality in the BigTen: https://github.com/juel15401/Big10UniversityCounties_COVID.git
COVID-19 human case data and SVI were provided from CDC (2021) and population data was provided from the U.S. Census Bureau (2019)

Field-specific reporting

Please select the one below that is the best fit for your research. If you are not sure, read the appropriate sections before making your selection.

☒ Life sciences ☐ Behavioural & social sciences ☐ Ecological, evolutionary & environmental sciences

For a reference copy of the document with all sections, see nature.com/documents/nr-reporting-summary-flat.pdf

Life sciences study design

All studies must disclose on these points even when the disclosure is negative.

Sample size	Sample size for the test validation was determined by FDA EUA guidelines. For all other analyses, sample was the entire surveillance population.
Data exclusions	No data were excluded from the population analysis. For the test validation, participants were excluded if they were tested more than 7 days after their most recent negative test.
Replication	For the development of covidSHIELD test, all experiments were performed more than three times with sufficient technical replicates for each experiment, as per US FDA guidelines. For analysis of acquired human specimen approved under IRB and IBC protocols, sample size are listed in Supplementary Tables 1, 4, and 7.
Randomization	All analyses are observational, so randomization was not possible
Blinding	Investigators were blinded to the results of the test validations while making inclusion decisions. Lab personnel were blinded to which samples were part of the validation study.

Reporting for specific materials, systems and methods

We require information from authors about some types of materials, experimental systems and methods used in many studies. Here, indicate whether each material, system or method listed is relevant to your study. If you are not sure if a list item applies to your research, read the appropriate section before selecting a response.

Materials & experimental systems

n/a	Involved in the study	
☒	☐	Antibodies
☒	☐	Eukaryotic cell lines
☒	☐	Palaeontology and archaeology
☒	☐	Animals and other organisms
☐	☒	Human research participants
☒	☐	Clinical data
☒	☐	Dual use research of concern

Methods

n/a	Involved in the study	
☒	☐	ChIP-seq
☒	☐	Flow cytometry
☒	☐	MRI-based neuroimaging

Human research participants

Policy information about studies involving human research participants

Population characteristics	All participants in the test validation were over 18, spoke English, were seeking a SARS-CoV-2 diagnostic test, and had tested negative for SARS-CoV-2 no more than 7 days previously. All participants in the surveillance program were associated with the University of Illinois Urbana-Champaign; no other demographic information is available for the sake of unidentifiability
Recruitment	Potential subjects at UIUC or UIC were approached by a member of the UIUC research team while waiting in line to have their COVID-19 test done per standard of care. Subjects at UW-Madison were identified during the course of their case investigation.<br>Biases are likely to be limited and to have no impact on results, as demographic characteristics have not been found to be related to test performance
Ethics oversight	All clinical samples from study participants were collected in accordance with Western IRB979 approved protocol number 20203538. Analysis of aggregate data from campus was ruled exempt by the University of Illinois Urbana-Champaign Institutional Review Board, protocol number 21216.

Note that full information on the approval of the study protocol must also be provided in the manuscript.
